# Acupoint injection for nonspecific chronic low back pain: A protocol of systematic review

**DOI:** 10.1097/MD.0000000000016478

**Published:** 2019-07-19

**Authors:** Jianglong Liao, Tao Wang, Wei Dong, Jingfan Yang, Jun Zhang, Lvyu Li, Jiankun Chen, Jian Li, Deguang Li, Yunxi Ma, Xiaoyan Zhang, Xiaoxia Tang, Bo Jiang, Ying Guo

**Affiliations:** Kunming Municipal Hospital of Traditional Chinese Medicine, Kunming, Yunnan, China.

**Keywords:** acupuncture injection therapy, low back pain, protocol, review

## Abstract

**Objective:**

The aim of this study was to evaluate the effect of acupuncture injection therapy for the patients with nonspecific chronic low back pain (CLBP) systematically.

**Methods:**

Four English online databases and 4 Chinese online databases will be researched systematically from their inception to December 31, 2018. Reference management software, Endnote X7, will be used to manage and screen the records. After removing the duplicate records, 2 independent reviewers will select the studies that meet the inclusion criteria. “Risk of table” recommend by Cochrane Handbook for Systematic Reviews of Interventions will be used to judge the quality of the included records. All data will be extracted by 1 reviewer and checked by another reviewer. Any disagree will be addressed via consulting a third reviewer in the above processes. Microsoft Excel will be used to manage and convert data if necessary. The missing data will be obtained via emailing the original authors of included studies. Review Manager (RevMan5.3) will be used to perform the data synthesis if enough data were collected. Otherwise, only the qualitative analysis will be performed. Based on the heterogeneity results, fixed-effect model or random-effect model will be used to estimate the overall effect of acupuncture injection therapy for patients with nonspecific CLBP. Meta-regression and subgroup analysis will be also performed to explore the sources of heterogeneity. If there are enough records included, the publish bias will be assessed by funnel plot. All procedures will be strictly performed in accordance with the Cochrane Handbook for Systematic Reviews of Interventions.

**Conclusion:**

This review will offer clinical evidence of acupuncture injection therapy for the patients with nonspecific CLBP.

**PROSPERO Research registration identifying number:**

CRD42019119158

## 1 Introduction

### 1.1 Description of condition

According to Global Burden of Disease Study (GBD) 2010, low back pain (LBP) was reported as the leading specific causes of years lived with disability (YLDs) in both developed and developing countries.^[1]^ It brings about 83.1 million YLDs loss, which is responsible for 10.7% of total YLDs.^[1]^ It also highlights as the major musculoskeletal (MSK) disorder because of high prevalence, high recurrence rate, and a greater disability, with sixth in terms of overall burden measured in disability-adjusted life years (DALYs) worldwide.^[2]^ LBP that is long-term >12 weeks was called chronic LBP (CLBP). Around 70% to 80% of adults believe that they have experienced CLBP sometime in their life.^[3]^ After the onset of CLBP, pain is the main symptom, and only 42% percent of patients were pain-free after 12 months.^[4]^ CLBP is also a primary cause of absent from work because of its mobility limitations, disability, and low quality of life.^[5]^ Obviously, CLBP has become an urgent global public health problem.

CLBP is not a specific term used to describe a disease but is a term used to describe a symptom related to pain occurring between the 12th rib and the inferior gluteal fold.^[6,7]^ It was reported that biophysical factors, comorbidities, and poor mental health were causes of LBP.^[8]^ However, in most cases with LBP, no cause can be found. That is, the LBP is “nonspecifc.”^[5,8,9]^

There are a range of different intervention strategies developed to relieve symptoms and improve function for the patients such as surgery, drug therapy, and exercise intervention.^[10]^ However, to date, it is difficult to offer the superiority of one approach over another.^[10,11]^ Therefore, there is an urgent need for a safe and effective treatment to relieve the symptoms of patients with nonspecific CLBP.

### 1.2 Description of Intervention

Acupuncture is widely used in the treatment of nonspecific CLBP^[12,13]^ and has been recommended by Clinical Guidelines of the American College of Physicians.^[14]^ On the basis of acupuncture therapy, a new form of acupuncture therapy intervention, acupuncture point (acupoint) injection, was developed to improve function and relieve the symptoms for the patients with nonspecific CLBP.^[15]^ Acupuncture point (acupoint) injection, which is also called pharmacoacupuncture, aqua acupuncture, water acupuncture, or herbal acupuncture, is widely used in traditional East Asian medicine, such as Korea and China.^[16]^ It combined therapy of acupuncture and medication. During pharmacoacupuncture, specific medication (such as vitamin, conventional medication, Chinese herbal extracts, among others) is injected into certain acupuncture points (acupoints) to treat diseases/conditions through synergetic effects of acupuncture and medication.^[17]^ Similar to traditional acupuncture therapy, acupuncture injection therapy works by stimulating the acupuncture points. Different from traditional acupuncture, pharmacoacupuncture injects also work through drugs injected into acupuncture point, which have antioxidant, microcirculatory, and neuroprotective effects. These medications were known to be beneficial for patients with nonspecific CLBP.^[18]^ It was reported that acupuncture point injection therapy enhanced and prolonged the effect of stimulation of acupuncture points^[18,19]^ and recommended that acupuncture point injection is convenient, simple, time-saving, and effective intervention.^[20]^ However, there is no systemic review to summarize and critically assess the efficacy of acupuncture point injection for nonspecific CLBP. Thus, this systemic review will be performed to solve this problem.

### 1.3 How the acupuncture injection therapy might work

The exact mechanism of acupuncture as a therapeutic method for the patients with nonspecific CLBP is unclear.^[21]^ However, there has been extensive research focused on the mechanisms related to its analgesic action. The clinical research evidence supported that acupuncture can significantly relieve the pain of patients with LBP compared with sham acupuncture or no acupuncture.^[21–24]^ Research shows that acupuncture can play a role in the pain relief through several mechanisms. First, acupuncture can regulate opioid release via inhibiting the dorsal horn to relieve pain.^[25]^ There are also some evidences that acupuncture may enhance analgesic effect via stimulating the production of acetylcholine and endorphins.^[26]^ Further evidence reported that analgesic effect of acupuncture may be related to the role of central neurotransmitters including the capicolaminas and cerotoninas.^[26]^ These neurotransmitters produce various effects, such as analgesic, muscle relaxant, anti-inflammatory.

Acupuncture point injection therapy is a combination of acupuncture and medication. Thus, it works through both acupuncture and medication for patients with nonspecific CLBP. Drugs that act as antioxidant or neuroprotectant, or Chinese herb extracts are injected into acupuncture point when acupuncture point injection.^[17]^ These medications have been demonstrated to benefit patients with nonspecific CLBP. Therefore, acupuncture can significantly relieve the pain and improve the function of patients and widely used for these patients.^[17]^

### 1.4 The importance of this review

LBP has become a global public health problem owing to its high prevalence, YLDs, and disability.^[27]^ There are many causes for LBP such as biophysical factors, comorbidities, and poor mental health.^[28]^ However, in most cases with LBP, no cause of the pain can be found. That is, the LBP is “nonspecific”. Many interventions were developed to relieve the pain and improve the function.^[11,29]^ However, there is no sufficient evidence indicated that any treatment is optimal.^[11,29]^ Acupuncture point injection therapy was used for patients with nonspecific CLBP widely. It combines the effects of traditional acupuncture and medicine. However, there is no study to systematically evaluate its clinical efficacy for patients with nonspecific CLBP. Therefore, this review is necessary to perform to systematically evaluate the safety and efficacy of acupuncture point injection therapy for the patients with nonspecific CLBP.

## 2 Methods

### 2.1 Design and registration

The protocol of this study was registered in the international prospective register of systematic reviews (https://www.crd.york.ac.uk/ PROSPERO/). Research registration unique identifying number is CRD42019119158. The Preferred Reporting Items for Systematic Reviews and Metaanalyses Protocols (PRISMA-P) statement was the guideline during the design of this study.^[30]^ Formal ethical approval of this systematic review is not applicable, as it is the secondary research of literature.

### 2.2 Selection criteria

#### 2.2.1 Type of participants

All participants must be adults older than 18years, suffering from nonspecific CLBP (persists for >12 weeks). We defined LBP as pain occurring between the 12th rib and the inferior gluteal fold in this study.^[8,9]^ It can include pain and neurological symptoms that radiate into one or both legs.^[5]^ Nonspecific LBP indicated that no specific cause was detectable, such as infection, neoplasm, metastasis, osteoporosis, rheumatoid arthritis, fracture, inflammatory process, or radicular syndrome.^[8,9]^

#### 2.2.2 Type of intervention

All clinical trials using acupuncture injection therapy as an intervention for nonspecific CLBP will be included in this study. The intervention of experimental group was acupuncture injection therapy which was defined as injection therapy at acupoints. There is no limitation on the medications injected such as vitamin, conventional medication, Chinese herbal extracts, or combinations of these medications. Control interventions such as rehabilitation training, surgery, exercise, only acupuncture, no intervention, placebo, or conventional medication (oral or injected at nonacupoint) will be accepted.

#### 2.2.3 Type of outcomes

Primary outcomes were defined as pain intensity (eg, usage of analgesic medications, visual analogue scale [VAS], and proportion of pain-free patients). Secondary outcomes included back pain-specific functional status, return to Work or Work Status (% of population, number of days of absenteeism), overall improvement Numeric Rating Scale (NRS), and function status measured by instruments such as modified Aberdeen LBP Scale, Owestry disability index (ODI), Roland-Morris Disability Questionnaire, lumbar flexibility (measured by fingertip to-floor distance).

#### 2.2.4 Type of studies

Only randomized controlled trials (RCTs) which compared acupuncture injection therapy with placebo or other therapy (included rehabilitation therapy, only acupuncture, injected at nonacupuncture point) will be included. The RCTs that injection therapy was used as one part of a multimodal treatment package will be excluded. The multimodal treatment package may challenge a valid summation of the clinical effects of acupuncture injection therapy for patients with non-specific CLBP. Non-randomized studies will be excluded.

#### 2.2.5 Search methods

##### 2.2.5.1 Online databases

Eight online databases will be searched from inception until December 31, 2018 with no language restrictions. Four English-language databases include EMBASE (via embase.com), Medline (via Pubmed), Cochrane Central Register of Controlled Trials (CENTRAL) in the Cochrane Library, and CINAHL (via EBSCOhost). Four Chinese-language databases are China National Knowledge Infrastructure (CNKI) database, Chinese Science and Technology Periodical (VIP) Database, Wanfang Database, and Sino-Med Database. The English terms were used individually or combined “acupoint injection,” “acupuncture point injection,” “hydroacupuncture,” “pharmacoacupuncture,” “low back pain,” “LBP,” “chronic low back pain,” and the Chinese searching terms were “xue wei zhu she (acupoint injection),” “shui zhen (acupoint injection),” “shu xue zhu she (acupoint injection).” The search strategies for each database were adjusted according to the characteristics of each individual database, and are listed in Table 1 (Medline via Pubmed).

**Table 1:**
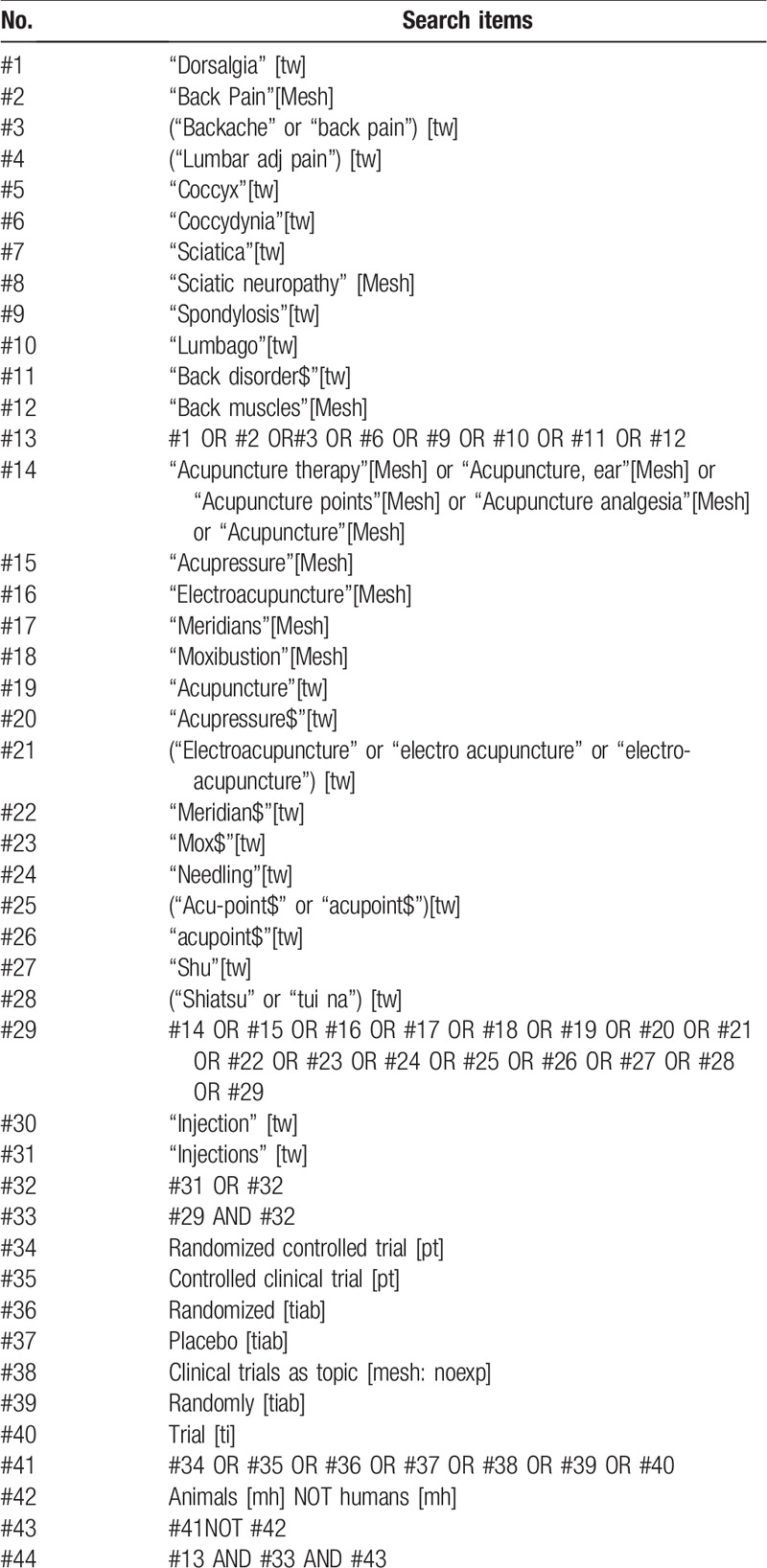
Search strategy for medline via pubmed.

##### 2.2.5.2 Other resources

For the non-English and non-Chinese language reports, we will also screen the abstracts if it is available in English or Chinese.

### 2.3 Data collection and analysis

#### 2.3.1 Literature screening

**Figure 1:**
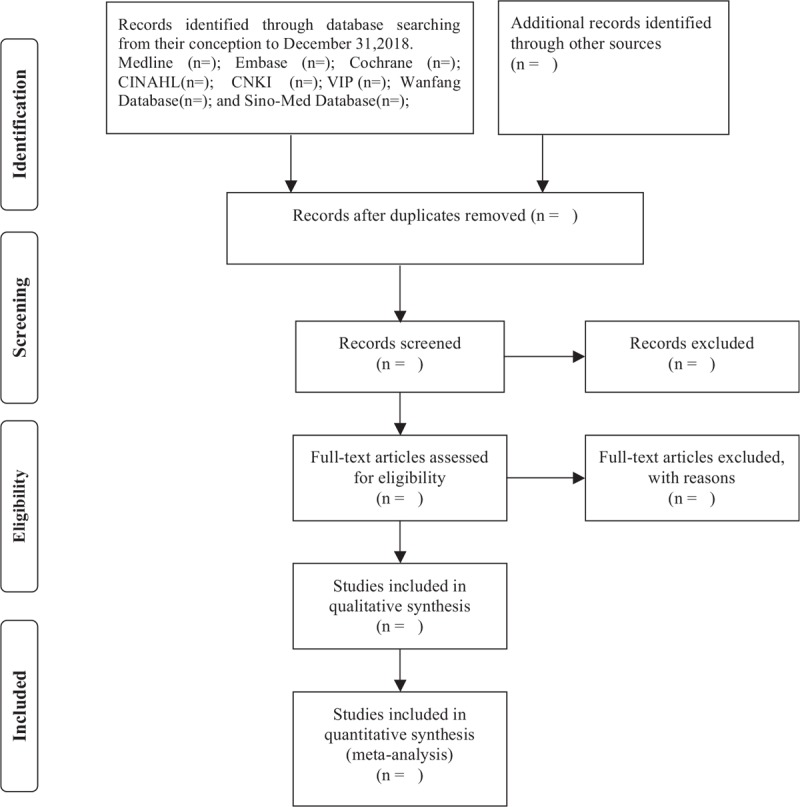
Flow diagram of the study selection process.

All records collected will be imported into the literature management software, EndNote X7. The duplicate records will be removed before 2 independent review authors screening all records. Potentially eligible studies and obviously irrelevant reports will be identified via reading the titles and abstracts of records. Qualified literature will be included after 2 independent review authors reading full text according to predetermined inclusion criteria. Qualified studies will be included depends on the type of patients, intervention, study design (PIS) strategy. If there is not enough information to make a judgment, we will contact the original author to get detail information. Any study that does not meet our inclusion criteria will be excluded. Any disagreements will be settled by consultation and discussion with a third reviewer. The PRISMA flow diagram (Fig. 1) will be used to report the details of the study selection process.

#### 2.3.2 Data extraction and management

All data will be extracted by one reviewer and checked by another reviewer. Five-part information data will be extracted in this review. The first part of information is basic information of studies including title, author, author institutions, the journal, publish year, among others. The second part of information extracted is characteristics of participants including age, sex, inclusion criteria, and exclusion criteria. The third information is the intervention of the experimental group and control group including the method, frequency, intensity, and period. The fourth part of information extracted is the design of the studies including the randomization, allocation concealment, and blinding. The last part of information is the outcomes and measurements including the outcome instruments, follow-up, drop-out, and adverse events. Missing data will be obtained by contacting the original author. The mean and standard deviation (SD) or standard error (SE) will be extracted for the measurement data. The number of events and the total number of the participants will be extracted for the enumeration data. For the studies that have >2 arms, only the 2 arms’ data that are most suitable for our review will be extracted. To perform the data conversion, spreadsheet software (Microsoft Excel) will be used to manage extracted data before entry to RevMan5.3. Any disagreement regarding data extraction will be discussed and consulted with a third experienced reviewer.

### 2.4 Methodological Quality assessment of included studies

Two reviewers will assess the quality of included studies independently using the “Risk of bias” (ROB) table which was recommend by Cochrane Handbook for Systematic Reviews of Interventions.^[31]^ The ROB table included 6 specific domains. Each domain is composed of >1 specific entries. There are 5 biases involved in this tool. The selection bias is related to 2 relevant domains (sequence generation and allocation concealment). The performance bias is related to 2 relevant domains (blinding of participants, personnel and outcome assessors; other potential threats to validity). The attrition bias is related 2 domains (incomplete outcome data and blinding of participants, personnel and outcome assessors, other potential threats to validity). The reporting bias was related to 1 domain (selective outcome reporting). These domains should be addressed in the tool by a single entry for each trial. “No” indicates high risk of bias in this domain. “Unclear” indicates unknown risk of bias or unclears. “Yes” indicates low risk of bias. Any inconsistencies should be resolved by discussing with a third reviewer.

### 2.5 Dealing with missing data

If there are missing data, we will email the original authors of each study to request any necessary data that is not reported in the manuscript. We will only analyze the available data when we fail to obtain the additional information. The potential impact of missing data on our review will be discussed in the discussion section. We emailed the authors of each study requesting any necessary data that were not comprehensively reported in the manuscript.

### 2.6 Evaluation of heterogeneity

The heterogeneity will be assessed by *χ*^2^ test (*P* < .1) and will be quantified by *I*^2^ statistic. When *I*^2^ value is >75%, we considered there is significant heterogeneity among the included studies according to Cochrane Handbook for Systematic Reviews of Interventions.^[31]^ The overall effect will be synthesized in a meta-analysis when there is a better homogeneity among the studies.^[32]^ Otherwise, meta-regression and subgroup analysis will be performed to explore the sources of heterogeneity.^[32]^

### 2.7 Evaluation of publication bias

Funnel plots will be used to evaluate the publication bias based on the number of included studies. To ensure the reliability of the results, funnel plots will only be generated when >10 trials were included in studies.^[32]^ Possible interpretations and language will been discussed in part of discussion.

### 2.8 Data synthesis

According to the heterogeneity results, the fixed-effect model or the random-effect model will be used for data synthesis. This step will be completed via Cochrane Review Manager (Revman5.3) recommend by Cochrane Handbook.^[31]^ If quantitative synthesis is not appropriate, only the qualitative analysis will be performed.

### 2.9 Subgroup analysis

If there are significant clinically and statistically heterogeneous (*I*^2^ > 75%) and enough RCT studies, subgroup analysis will be carried out. According to the characteristics of measurement tools (ie, VAS, NRS), the origins of heterogeneity will be carried out.

### 2.10 Sensitivity analysis

To identify the influence of trials with a low methodological quality on results, sensitivity analysis will be performed. If the pooled effective size changed significantly after excluding a study, we consider this study influences the results. The results of the sensitivity analysis will be discussed in part of discussion and conclusion.

### 2.11 Grading the quality of evidence

The Grading of Recommendations Assessment, Development and Evaluation (GRADE) system will be used to evaluate the quality of evidence for outcomes which rate the quality as very low, low, moderate, or high levels.^[33]^

## 3 Discussion

LBP is an extremely common symptom in populations worldwide and occurs in all age groups, from children to the elderly population.^[4,34,35]^ The LBP that lasts >3 months was defined as CLBP. Normally, a specific cause of LBP is rarely can be confirmed. Therefore, most LBP is termed nonspecific. Numerous interventions have been used to relieve pain and improve function for patients with LBP. However, there is no optimal treatment.^[10,11]^ Acupuncture injection therapy was reported as one of effective treatments for patients with nonspecific CLBP and used widely in Southeast Asian countries.^[16]^ It was shown that water acupuncture is more effective than traditional acupuncture because it combines the traditional acupuncture and medication. But there is no systematic and comprehensive evaluation of its efficacy. Therefore, it is urgently necessary to evaluate the effect of water acupuncture therapy for the patients with nonspecific CLBP.

In our opinion, this review may provide systemic and comprehensive evaluation for the effect of acupuncture point injection for the patients with nonspecific CLBP. Such a systematic review will also give help to make decisions regarding future practice of water acupuncture therapy for nonspecific CLBP relief and function improvement.

The advantage of this review is that it was designed in strict accordance with Cochrane Handbook for Systematic Reviews of Interventions. Good methodological design makes research results reliable and credible. However, there are also some limitations we should note. The first source of variation of water acupuncture therapy is the different acupuncture point used among included studies. Second, pharmacological agent injected may differ from study to study which also a source of the clinical heterogeneity. Third, diverse control settings may also lead to the presence of heterogeneity. Moreover, the diversity of duration, and dosage of acupuncture point injection may result in a significant heterogeneity among the included studies.

## Acknowledgments

The authors acknowledge Jing Li and her team at Chinese Cochrane Centre for their design assistance.

## Author contributions

**Conceptualization:** Bo Jiang, Ying Guo.

**Funding acquisition:** Tao Wang.

**Investigation:** Jiankun Chen, Jian Li, Deguang Li, Yunxi Ma, Xiaoyan Zhang, Xiaoxia Tang.

**Methodology:** Wei Dong, Jingfan Yang, Jun Zhang, Xiaoyan Zhang, Xiaoxia Tang.

**Resources:** Lvyu Li.

**Writing – original draft:** Jianglong Liao, Tao Wang.

**Writing – review & editing:** Jianglong Liao, Tao Wang, Wei Dong, Jingfan Yang, Jun Zhang, Lvyu Li, Deguang Li, Yunxi Ma, Xiaoyan Zhang, Xiaoxia Tang, Bo Jiang.

## References

[1] Vos T Flaxman AD Naghavi M Years lived with disability (YLDs) for 1160 sequelae of 289 diseases and injuries 1990-2010: a systematic analysis for the Global Burden of Disease Study 2010. Lancet 2012;380:2163–96. 23245607 10.1016/S0140-6736(12)61729-2 PMC6350784

[2] Damian H Lyn M Peter B The global burden of low back pain: estimates from the Global Burden of Disease 2010 study. Ann Rheum Dis 2014;73:975–81. 24665117 10.1136/annrheumdis-2013-204631

[3] Crombez G Vlaeyen JWS Heuts PHTG Pain-related fear is more disabling than pain itself: evidence on the role of pain-related fear in chronic back pain disability. Pain 1999;80:329–39. 10204746 10.1016/s0304-3959(98)00229-2

[4] Hoy D Bain C Williams G A systematic review of the global prevalence of low back pain. Arthritis Rheum 2014;64:2028–37. 10.1002/art.34347 22231424

[5] Buchbinder R Hartvigsen J Dan C What low back pain is and why we need to pay attention. Lancet 2018;391:2356–67. 29573870 10.1016/S0140-6736(18)30480-X

[6] Van Zundert J Hans G van Kuijk S Low back pain. Lancet 2018;392:2548–9. 10.1016/S0140-6736(18)33124-6 30563636

[7] Hartvigsen J Hancock MJ Kongsted A What low back pain is and why we need to pay attention. Lancet 2018;391:2356–67. 29573870 10.1016/S0140-6736(18)30480-X

[8] Buchbinder R van Tulder M Öberg B Low back pain: a call for action. Lancet 2018;391:2384–8. 29573871 10.1016/S0140-6736(18)30488-4

[9] Maher C Underwood M Buchbinder R Non-specific low back pain. Lancet 2017;389:736–47. 27745712 10.1016/S0140-6736(16)30970-9

[10] Nascimento P Costa LOP Araujo AC Effectiveness of interventions for non-specific low back pain in older adults. A systematic review and meta-analysis. Physiotherapy 2019;105:147–62. 30563712 10.1016/j.physio.2018.11.004

[11] Buchbinder R Hartvigsen J Dan C Prevention and treatment of low back pain: evidence, challenges, and promising directions. Lancet 2018;391:2368–83. 29573872 10.1016/S0140-6736(18)30489-6

[12] Xu M Yan S Yin X Acupuncture for chronic low back pain in long-term follow-up: a meta-analysis of 13 randomized controlled trials. Am J Chin Med 2013;41:1–9. 23336503 10.1142/S0192415X13500018

[13] Hutchinson AJ Ball S Andrews JC The effectiveness of acupuncture in treating chronic non-specific low back pain: a systematic review of the literature. J Orthop Surg Res 2012;7:36–44. 23111099 10.1186/1749-799X-7-36 PMC3563482

[14] Qaseem A Wilt TJ Mclean RM Noninvasive treatments for acute, subacute, and chronic low back pain: a clinical practice guideline from the American College of Physicians. Ann Intern Med 2017;166:514–30. 28192789 10.7326/M16-2367

[15] Bin Z Treating discogenic lower back pain by acupuncture. Clin J Chin Med 2015 38–9.

[16] Strudwick MW Hinks RC Choy STB Point injection as an alternative acupuncture technique – an exploratory study of responses in healthy subjects. Acupunct Med 2007;25:166–74. 18160927 10.1136/aim.25.4.166

[17] Kun a Acupuncture Injection Therapy. Beijing: China Medical Science Press; 2012.

[18] MarkWStrudwick Roderick C Hinks Point injection as an alternative acupuncture technique—an exploratory study of responses in healthy subjects. Acupunct Med 2007;25:166–74. 18160927 10.1136/aim.25.4.166

[19] Wang L Cardini F Zhao W Vitamin K acupuncture pint injection for severe primary dysmenorrhea: an international pilot study. MedGenMed 2004;6:45–145. PMC1480551 15775872

[20] Yang LC Jawan B Chen CN Comparison of P6 acupoint injection with 50% glucose in water and intravenous droperidol for prevention of vomiting after gynecological laparoscopy. Acta Anaesthesiol Scand 2010;37:192–4. 10.1111/j.1399-6576.1993.tb03699.x 8447210

[21] Fan AY Ouyang H Qian X Discussions on real-world acupuncture treatments for chronic low-back pain in older adults. J Integr Med 2019;2:71–6. 10.1016/j.joim.2019.01.005 30738771

[22] Megan L Philip C Effectiveness of acupuncture for nonspecific chronic low back pain: a systematic review and meta-analysis. Spine 2013;38:2124–38. 24026151 10.1097/01.brs.0000435025.65564.b7

[23] Yu-Jeong C Yun-Kyung S Yun-Yeop C Acupuncture for chronic low back pain: a multicenter, randomized, patient-assessor blind, sham-controlled clinical trial. Spine 2013;38:549–57. 23026870 10.1097/BRS.0b013e318275e601

[24] Chao HY Suen KP Shen J Changes in sleep with auricular point acupressure for chronic low back pain. Behav Sleep Med 2016;14:279–94. 26244591 10.1080/15402002.2014.981820

[25] Ruan H Li X Cai W [Effect of 5-HT and somatostatin on SP and chronic pain initiated electrical activity of neurons in spinal dorsal horn]. Zhen Ci Yan Jiu 1996;21:27–31. 9387337

[26] Kwokming James C Neurobiological mechanisms of acupuncture for some common illnesses: a clinician's perspective. J Acupunct Meridian Stud 2014;7:105–14. 24929454 10.1016/j.jams.2013.07.008

[27] March L Smith EU Hoy DG Burden of disability due to musculoskeletal (MSK) disorders. Best Pract Res Clinical Rheumatol 2014;28:353–66. 25481420 10.1016/j.berh.2014.08.002

[28] Edit V Eva S Maria K Psychosocial, educational, and somatic factors in chronic nonspecific low back pain. Rheumatol Int 2013;33:587–92. 22476243 10.1007/s00296-012-2398-0

[29] Casser HR Seddigh S Rauschmann M Acute lumbar back pain. Dtsch Arztebl Int 2016;113:223–34. 27120496 10.3238/arztebl.2016.0223 PMC4857557

[30] Shamseer L Moher D Clarke M Preferred reporting items for systematic review and meta-analysis protocols (PRISMA-P) 2015: elaboration and explanation. BMJ 2015;350:g7647–72. 25555855 10.1136/bmj.g7647

[31] Higgins. JPT GS. Cochrane Handbook for Systematic Reviews of Interventions Version 5.1.0. 2011; Available at: https://www.cochrane.org/search/manuals/handbook?manual=Handbook Accessed September 4, 2018.

[32] Copas J Shi JQ Meta-analysis, funnel plots and sensitivity analysis. Biostatistics 2000;1:247–62. 12933507 10.1093/biostatistics/1.3.247

[33] Guyatt GH Oxman AD Vist GE GRADE: an emerging consensus on rating quality of evidence and strength of recommendations. BMJ 2008;336:924–6. 18436948 10.1136/bmj.39489.470347.AD PMC2335261

[34] Kamper SJ Henschke N Hestbaek L Musculoskeletal pain in children and adolescents. Braz J Phys Ther 2016;20:275–84. 27437719 10.1590/bjpt-rbf.2014.0149 PMC4946844

[35] Hartvigsen J Christensen K Frederiksen H Back pain remains a common symptom in old age. A population-based study of 4486 Danish twins aged 70-102. Eur Spine J 2003;12:528–34. 12748896 10.1007/s00586-003-0542-y PMC3468008

