# Acupoint injection for nonspecific chronic low back pain: A protocol of systematic review: Erratum

**DOI:** 10.1097/MD.0000000000018084

**Published:** 2019-11-11

In the article, “Acupoint injection for nonspecific chronic low back pain: A protocol of systematic review”,^[1]^ which appears in Volume 98, Issue 29 of *Medicine*, some of the details in Table 1 appear incorrectly. The correct Table 1 is:

**Table 1:**
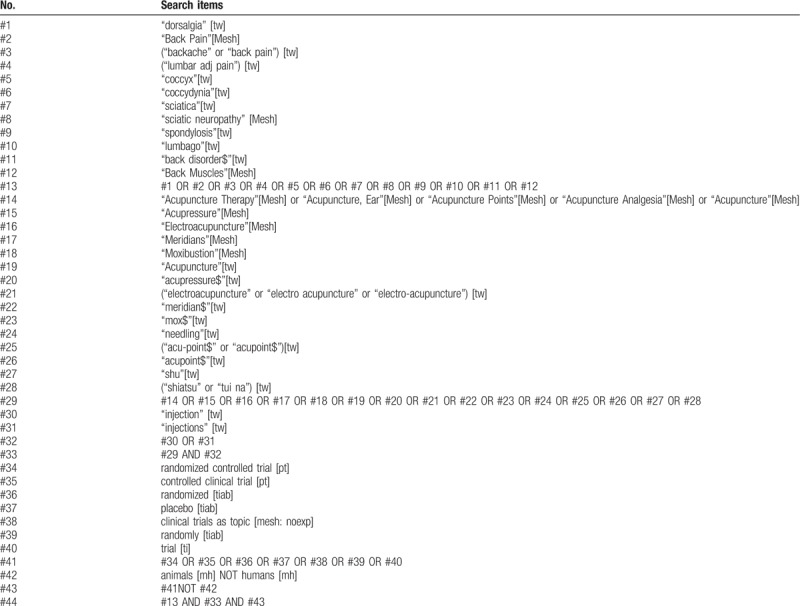
Search strategy for medline via pubmed.
